# Melatonin ameliorates inflammation and improves outcomes of ischemia/reperfusion injury in patients undergoing coronary artery bypass grafting surgery: a randomized placebo-controlled study

**DOI:** 10.1007/s10495-024-02040-6

**Published:** 2024-12-04

**Authors:** Eman Ahmed Casper, Lamia El Wakeel, Nagwa A. Sabri, Ramy Khorshid, Mohamed A. Gamal, Sarah F. Fahmy

**Affiliations:** 1 Department of Clinical Pharmacy, Faculty of Pharmacy, Ain Shams University, Ankara Street, Sheraton buildings, Cairo, 11566 Egypt; 2 Department of Cardiovascular and Thoracic Surgery, Ain Shams University Hospital, Faculty of Medicine, Ain Shams University, Cairo, Egypt

**Keywords:** Melatonin, CABG, Ischemia/reperfusion injury, NF-kB, IL-6, TNF-α, Troponins

## Abstract

To investigate the protective role of high dose melatonin concerning myocardial I/R injury and inflammation in patients undergoing on-pump coronary artery bypass grafting (CABG) surgery by evaluating IR/inflammatory biomarkers and clinical outcomes. This was a prospective; randomized; single-blinded placebo-controlled study conducted at cardio-thoracic surgery department of the Academy of the Cardiovascular and Thoracic Surgery, Ain Shams University. Eligible patients were randomly allocated to; melatonin-treated group (MTG) or placebo-treated group (PTG). The MTG (*n* = 17) received 60 mg/day melatonin capsules daily starting 5 days before surgery in addition to the standard of care. PTG (*n* = 17) received placebo also 5 days before surgery plus standard of care. The levels of nuclear factor kappa beta (NF-κb) (primary outcome), tumor necrosis factor (TNF-α), cardiac troponins I, and IL-6 levels were all assessed for both groups at five time points: baseline before melatonin or placebo administration (T0), before cross-clamp application(T1), 5 min after cross-clamp removal(T2), 6 h after cross-clamp removal(T3) and 24 h after cross-clamp removal(T4). Blood pressure was assessed at baseline, pre-operative and 24-hours post-operative. The Quality of recovery-40 score (QOR-40) was assessed for both groups on day 4 after surgery. TNF-α levels decreased in the MTG at T1(*p* = 0.034) versus PTG. At T2(*p* = 0.005), and T3(*p* = 0.04), TNF-α significantly increased in PTG versus MTG. Troponins significantly increased in PTG at T3 (*p* = 0.04) versus MTG. NF-κB levels declined at T1 (*p* = 0.013) and T2 (*p* = 0.0001) in MTG compared to PTG. IL-6 significantly increased in PTG versus MTG at T3 (*p* = 0.04). The QOR-40 score significantly decreased in MTG versus PTG. MTG had statistically significant decrease in DBP compared to the placebo group (*p* = 0.024). MTG had a statistically significant shorter intubation time than did the placebo group (*p* = 0.03). Melatonin 60 mg was well-tolerated without any reported side effects. Our findings suggested that melatonin could ameliorate myocardial I/R injury after on-pump CABG and that this outcome was essentially correlated to its antiapoptotic and anti-inflammatory effects. Trial registration: ClinicalTrials.gov registration number NCT05552586, 9/2022.

## Introduction

According to the World Health Organization (WHO), cardiovascular diseases (CVDs) are considered the leading cause of death worldwide [1]. Coronary artery disease (CAD) is considered one of the most common cardiovascular diseases [2]. Coronary artery bypass grafting surgery (CABG) is indicated for the treatment of coronary artery disease patients with high risk features [3]. One of the most devastating complications of coronary artery bypass graft (CABG) is the myocardial ischemic-reperfusion injury through cardioplegic arrest. The reperfusion process helps to salvage the ischemic myocardium however, this process can lead to an extensive inflammatory response and serious postoperative complications [4]. It releases the enzyme troponin and induces myocyte death. In acute cases, reperfusion arrhythmias can cause ventricular tachycardia and ventricular fibrillation, premature ventricular contractions, and even post-reperfusion sudden cardiac death [5].

The pathophysiology of ischemia-reperfusion injury is multifactorial. During the reperfusion period, free radical scavenging mechanisms are impaired due to the overwhelming release of oxygen free radicals. Therefore, reactive nitrogen species and reactive oxygen species accumulate, causing further myocardial damage. This makes the myocardium prone to inflammation and oxidative stress. Free radicals also change calcium signaling in platelets and promote platelet aggregation [5, 6].

Inflammation is another player in the progression of CAD. Nuclear factor-kappa B (NF-κB) is a major regulator of the inflammatory response by regulating the gene expression of inflammatory cytokines which are regarded as major contributors to the development of cardiovascular diseases in humans [7]. Different inducers can activate NF-κB, including reactive oxygen species and inflammatory cytokines [8].

When activated, NF-kB increases the levels of inflammatory markers such as tumor necrosis factor-alpha (TNF-α), C reactive protein (CRP), interleukin-1beta (IL-1β), interleukin-6 (IL-6), and interferon-gamma (IFN-γ) [9, 10].

Despite innovations in reperfusion strategies, including advanced surgical equipment, and the use of antiplatelet and antithrombotic agents, there is still no effective therapy for preventing myocardial ischemia-reperfusion injury [11].

Research on the role of oxidative stress and inflammation in the pathophysiology of post-CABG complications has accumulated. Consequently, several studies have tested various agents before or during surgery with the aim of reducing post-CABG complications [12].

Melatonin is a product of the pineal gland that acts as a regulator of the circadian rhythm [13]. In addition to its role in regulating the circadian rhythm, melatonin exerts several effects including free radical scavenging and ameliorating oxidative stress and inflammation [4]. These previously investigated actions have highlighted the possible cardio-protective effects of melatonin [14]. Moreover, melatonin receptors are present in the vascular structure of the human body which may contribute to their effects on various sites of the body [15].

The relationship between coronary artery disease severity and plasma levels of melatonin has been previously described in several studies [15, 16].

Since melatonin levels decline progressively over the lifespan, the loss of melatonin during aging could contribute to a gradually increased predisposition to hypertension, arrhythmias, and coronary artery diseases [17, 18]. In coronary artery disease patients, night-time 6-hydroxymelatonin sulfate was significantly decreased, indicating decreased pineal melatonin production [19].

Studies on patients undergoing CABG have revealed that patients with low postoperative melatonin levels in the blood are more prone to developing complications, while prophylactic use of melatonin is linked to lower levels of cardiac biomarkers such as troponin I and CK-MB [20]. Several studies have investigated the potential role of melatonin in reducing oxidative stress and inflammation in CABG patients based on its antioxidative and anti-inflammatory effects, with promising results and with no reported significant side effects [4, 21–24]. However, no well-controlled clinical trials have been carried out to test the effect of high-dose melatonin (60 mg) on inflammation and clinical outcomes in CABG patients. Given that melatonin has low intestinal absorption and is safe even at very high doses (300 mg/kg) [25], we aimed to conduct the current clinical trial to investigate the effects of high dose melatonin (60 mg) administration to CABG patients and its impact on clinical outcomes, as well as on markers of apoptosis and inflammation.

## Results

**Fig. 1:**
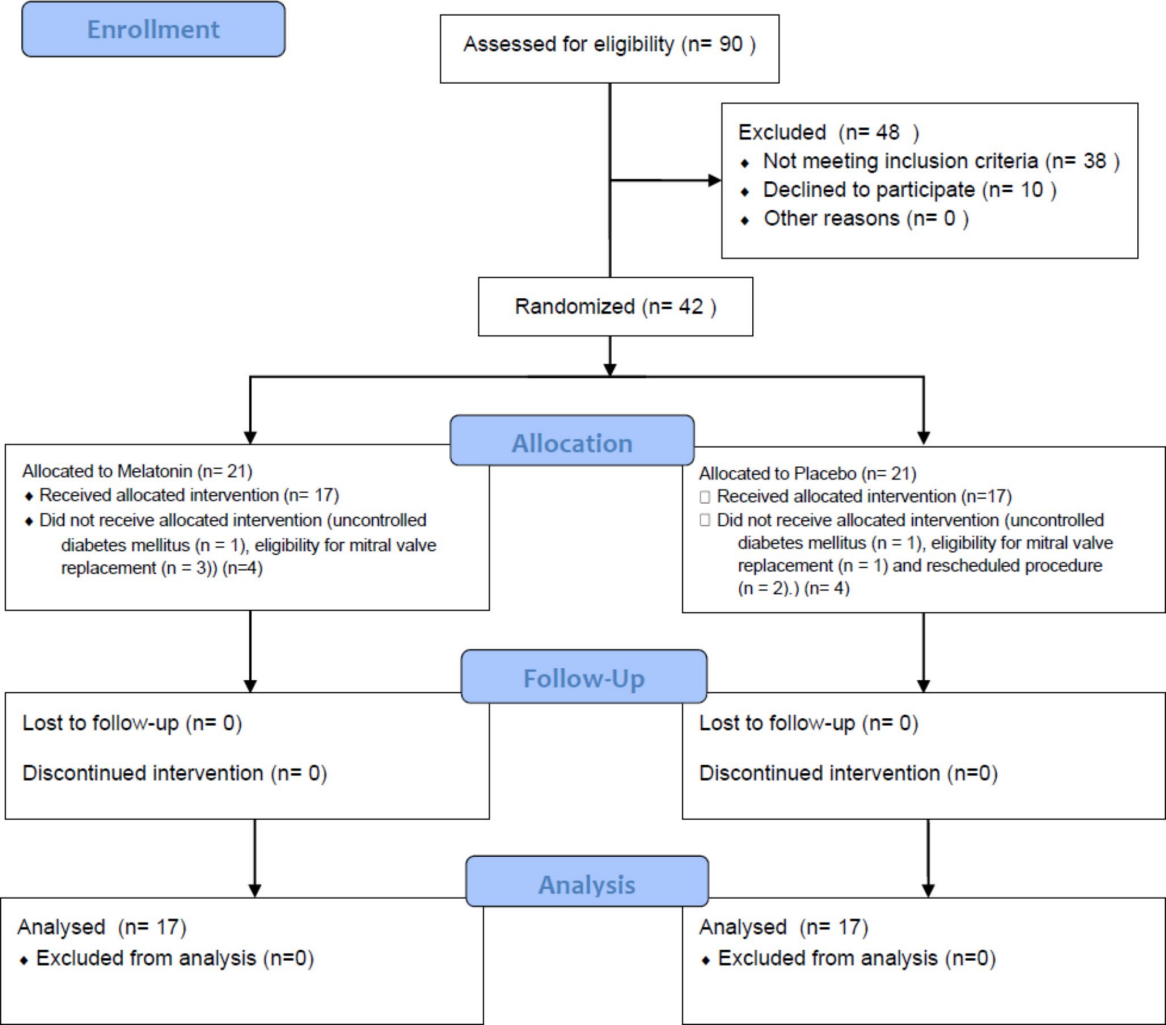
Consort Study flow diagram

Out of 90 patients screened, only 42 fulfilled the inclusion criteria and were recruited for the study. Out of the 42 patients, only 34 completed the study and 8 patients were excluded for various reasons; had uncontrolled diabetes mellitus (*n* = 2), were eligible for mitral valve replacement (*n* = 4), or underwent rescheduled procedure (*n* = 2). Thirty-four patients were allocated to either the PTG (*n* = 17) or the MTG (*n* = 17) (Fig. 1).

### Baseline evaluation

At baseline, there was no significant difference between the two study groups in terms of patient demographics, routine therapy used, risk factors for cardiovascular disease, vital parameters, baseline laboratory or Society of Thoracic Surgeons risk score (Tables 1, 2, 3 and 4).

**Table 1:** Demographic data and risk factors for cardiovascular diseases for both groups

Variable	MTG group (*n* = 17)	PTG group (*n* = 17)	*P*-value
Demographics			
Gender			
Male	14 (82.3%)	12 (70.5%)	0.69 ^**a**^
Female	3 (17.6%)	5 (29.5%)	
Age (years) Mean ± SD	56.6 ± 8.031	51.8 ± 7.83	0.76 ^**b**^
BMI (Kg/m^2^) Mean ± SD	31.59 ± 3.68	31.41 ± 5.89	0.92 ^**b**^
Risk Factors			
Smoking status			
Smoker Ex-smoker Second-hand smoking Nonsmoker	8 (47%) 6 (35.2%) 1 (0.05%) 2 (11.7%)	9 (52.9%) 2 (11.7%) 3 (17.6%) 3 (17.6%)	0.35^a^
Hypertension			
Yes No	9 (52.9%) 8 (47.1%)	10 (58.8%) 7 (41.2%)	1^**a**^
Dyslipidemia			
Yes No	12 (70.58%) 5 (29.42%)	14 (82.35%) 3 (17.64%)	0.69^**a**^
Family history			
Yes No	8 (47.1%) 9 (52.9%)	11 (64.7%) 6 (35.3%)	0.49^**a**^
Obesity			
Yes No	17 (100%) 0 (0%)	16 (94.11%) 1 (5.88%)	1^**a**^
Diabetes			
Yes No	8 (47.1%) 9 (52.9%)	11 (64.7%) 6 (35.3%)	0.73^**a**^
No. of non-cardiac surgeries Yes No	9 (52.9%) 8 (47.1%)	11 (64.7%) 6 (35.3%)	0.73 ^**a**^
No. of cardiac surgeries Yes No	6 (35.3%) 11 (64.7%)	7 (41.1%) 10 (58.8%)	1 ^**a**^
Coronary heart disease duration (months) Median ± IQR	11 (5–54)	8 (3–54)	0.46 ^**c**^
STS predicted Morbidity and Mortality (%) Median ± IQR	3.94 (2.87–5.135)	3.62 (2.97–5.35)	0.97 ^**c**^

PTG group: the patient group that received placebo

MTG group: the patient group that received melatonin 60 mg

MTG: Melatonin Treated group; PTG: Placebo-treated group; STS: Society of Thoracic Surgeons

^a^ Statistical test: Fisher’s Exact test

^b^ Statistical test: Unpaired t-test test

^c^ Statistical test: Mann-Whitney test

Statistical significance (*P* < 0.05)

**Table 2:** Prescribed medications in both groups

Variable		MTG group (*n* = 17)		PTG group (*n* = 17)		P-value
		No	%	No	%	
Beta-blockers	Yes	16	94.1	15	88.2	1 ^a^
	No	1	5.8	2	11.7	
Aspirin	Yes	17	100	17	100	-
	No	0	0	0	0	
Clopidogrel	Yes	17	100	17	100	-
	No	0	0	0	0	
ACEI/ARB	Yes	11	64.7	12	70.5	1 ^a^
	No	6	35.3	5	29.5	
CCB	Yes	1	5.8	1	5.8	0.6 ^a^
	No	16	94.1	16	94.2	
Nitrates	Yes	11	64.7	12	70.5	1 ^a^
	No	6	35.3	5	29.5	
Statins	Yes	15	88.2	15	88.2	1 ^a^
	No	2	11.8	2	11.8	
Aldosterone antagonist	Yes	3	17.6	1	5.8	1 ^a^
	No	14	82.4	16	94.2	
Sublingual nitrates	Yes	9	52.9	7	41.2	0.73 ^a^
	No	8	47.1	10	58.8	

PTG group: the patient group that received placebo

MTG group: the patient group that received melatonin 60 mg

ACEI: Angiotensin Converting Enzyme Inhibitor; ARB: Angiotensin Receptor Blocker; CCB: Calcium Channel Blocker; MTG: Melatonin Treated group; PTG: Placebo-treated Group

^a^ Statistical test: Fisher’s Exact test

Statistical significance (*P* < 0.05)

**Table 3:** Baseline, pre-operative, and 24-hour post-operative vital parameters in both groups

Variable		MTG group (*n* = 17)	PTG group (*n* = 17)	*P*-value
Heart rate (BPM) (Median ± IQR)	Baseline	75 (70–85)	77 (71–79)	1 ^a^
	Pre-operative	77 (70–80)	80 (60–91)	0.92 ^a^
	24 h Post-operative	80 (65–99)	85 (76–98)	0.9 ^a^
	P-value	0.07 ^b^	0.043* ^b^	
Oxygen Saturation (%) (Median ± IQR)	Baseline	97 (96–98)	99 (95–99)	0.23 ^a^
	Pre-operative	98 (97–99)	99 (96–99)	0.17 ^a^
	24 h Post-operative	100 (97–100)	99 (95–100)	0.18 ^a^
	P-value	0.08 ^**b**^	0.11 ^**b**^	
DBP (mmHg) (Median ± IQR)	Baseline	80 (70–80)	70 (70–80)	0.62 ^a^
	Pre-operative	70 (64–80)	70 (70–80)	1 ^a^
	24 h Post-operative	60 (60–70)	75 (62.5–80)	0.024* ^a^
	P-value	0.0001* ^**b**^	0.88 ^b^	
SBP (mmHg) (Median ± IQR)	Baseline	120 (110–140)	115 (110–130)	0.87^a^
	Pre-operative	120 (110–140)	120 (110–135)	1 ^a^
	24 h Post-operative	110 (105–125)	130 (112–140)	0.087 ^a^
	P-value	0.05 ^b^	0.44 ^b^	

PTG group: the patient group that received placebo

MTG group: the patient group that received melatonin 60 mg

BPM: Beat Per Minute; DBP: Diastolic Blood Pressure; MTG: Melatonin Treated Group; PTG: Placebo-treated group; SBP: Systolic Blood Pressure

^a^ Statistical test: Mann-Whitney test

^b^ Statistical test: Friedman’s test

Statistical significance (*P* < 0.05)

**Table 4:** Baseline and end-of-study laboratory parameters for the two groups

Variable	Time	MTG group (*n* = 17)	PTG group (*n* = 17)	*p*-value
Total Cholesterol (mg/dl)	Baseline (Mean ± SD)	156.2 ± 50.18	148.9 ± 35.87	1 ^**a**^
	24-hour after cross-clamp removal (Mean ± SD)	150.3 ± 47.32	138.3 ± 35.21	0.41 ^**a**^
	%change from baseline (Median ± IQR)	-0.03((-0.05) - (-0.02))	-0.02((-0.07) − 0.02)	0.26 ^**b**^
LDL (mg/dl)	Baseline (Mean ± SD)	85.35 ± 40.57	78.65 ± 35.22	0.61 ^**a**^
	24-hour after cross-clamp removal (Mean ± SD)	80.94 ± 38.15	72.11 ± 29.56	0.47 ^**a**^
	%change from baseline (Median ± IQR)	-0.05((-0.09)-( -0.007))	-0.05 (-0.16–0.07)	0.89 ^**b**^
HDL (mg/dl)	Baseline (Mean ± SD)	41.18 ± 6.09	41.71 ± 8.26	0.83 ^**a**^
	24-hour after cross-clamp removal (Mean ± SD)	40.82 ± 6.46	42.25 ± 5.23	0.49 ^**a**^
	%change from baseline (Mean ± SD)	-0.01 ± 0.09	0.008 ± 0.18	0.66 ^**a**^
Triglycerides (mg/dl)	Baseline (Median ± IQR)	111 (94–150)	135 (111–147)	0.64 ^**b**^
	24-hour after cross-clamp removal (Median ± IQR)	105 (89–147)	115 (95–140)	0.97 ^**b**^
	%change from baseline (Median ± IQR)	-0.02 (-0.07–0.01)	-0.03 (-0.07–0.03)	0.98 ^**b**^
Serum creatinine (mg/dl)	Baseline (Mean ± SD)	1.08 ± 0.24	1.02 ± 0.23	0.51 ^**a**^
	24-hour after cross-clamp removal (Mean ± SD)	1.23 ± 0.37	1.05 ± 0.28	0.12 ^**a**^
	%change from baseline (Mean ± SD)	0.31 ± 0.56	0.04 ± 0.47	0.13 ^**a**^
Blood Urea Nitrogen (mg/dl)	Baseline (Median ± IQR)	30 (20.5–41.5)	25 (22.5–40.5)	0.58 ^**b**^
	24-hour after cross-clamp removal (Median ± IQR)	37 (25–41.5)	34.5 (24–46.75)	0.93 ^**b**^
	%change from baseline (Median ± IQR)	0.09 (-0.09–0.25)	0.11 (-0.08–0.35)	0.69 ^**b**^
Sodium (mmol/l)	Baseline (Median ± IQR)	137 (135–142)	137 (136–141)	1 ^**b**^
	24-hour after cross-clamp removal (Median ± IQR)	143 (140–148)	146 (140.3–149)	0.39 ^**b**^
	%change from baseline (Median ± IQR)	0.04 ( 0.01–0.06)	0.05 (0.02–0.08)	0.36 ^**b**^
Potassium (mmol/l)	Baseline (Mean ± SD)	4.17 ± 0.52	4.41 ± 0.63	0.23 ^**a**^
	24-hour after cross-clamp removal (Mean ± SD)	4.1 ± 0.43	4.13 ± 0.62	0.87 ^**a**^
	%change from baseline (Mean ± SD)	-0.03 ± 0.17	-0.09 ± 0.23	0.36 ^**a**^
Blood glucose (mg/dl)	Baseline (Mean ± SD)	158.4 ± 33.01	148.1 ± 43.61	0.44 ^**a**^
	24-hour after cross-clamp removal (Mean ± SD)	134 ± 22.31	172.1 ± 36.97	**0.002** ^**a**^
	%change from baseline (Median ± IQR)	-0.13 (-0.23–0.02)	0.09 (0.03–0.22)	**0.002** ^**b**^
Hemoglobin (g/dL)	Baseline (Mean ± SD)	13.41 ± 1.56	13.25 ± 1.48	0.76 ^**a**^
	24-hour after cross-clamp removal (Median ± IQR)	10.2 (10–12.1)	10.45 (10.03–11.98)	0.48 ^**b**^
	%change from baseline (Mean ± SD)	-0.24 ± 0.19	-0.21 ± 0.16	0.64 ^**a**^
Platelets count (x10^3)	Baseline (Mean ± SD)	256 ± 72.42	230.8 ± 61.37	0.28 ^**a**^
	24-hour after cross-clamp removal (Mean ± SD)	188.4 ± 67.59	198.8 ± 48.09	0.62 ^**a**^
	%change from baseline (Median ± IQR)	-0.31 (-0.63- -0.18)	-0.09 (-0.38- -0.02)	0.06 ^**b**^
PT (seconds)	Baseline (Median ± IQR)	13.6 (12.1–14.5)	13.8 (12.35–14.4)	0.67 ^**b**^
	24-hour after cross-clamp removal (Median ± IQR)	15.8 (14.8–16.9)	15 (14–15.5)	0.27 ^**b**^
	%change from baseline (Median ± IQR)	0.17 (0.03–0.21)	0.09 (-0.06–0.16)	0.11 ^**b**^

PTG group: the patient group that received placebo

MTG group: the patient group that received melatonin 60 mg

HDL: High-Density Lipoprotein; LDL: Low-Density Lipoprotein; MTG: Melatonin Treated group; PTG: Placebo-treated group PT: Prothrombin Time

^a^ Statistical test: Unpaired t-test

^b^ Statistical test: Mann-Whitney test

Statistical significance (*P* < 0.05)

None of the domains of the Amsterdam Preoperative Anxiety and Information Scale (APAIS) were statistically significant different between the two groups (Table 5).

**Table 5:** Scores of Medication adherence, anxiety and quality of Recovery questionnaires in both groups

Variable		MTG group (*n* = 17)	PTG group (*n* = 17)	*P*-value
VAS (Mean ± SD)		63.53 **±** 22.06	61.18 ± 22.88	0.76 ^**a**^
MMAS (Mean ± SD)		7.34 **±** 0.95	7.56 ± 0.69	0.44 ^**a**^
APAIS (Mean ± SD)	Anesthesia-related Anxiety	6.41 ± 2.48	6.35 ± 2.32	0.94 ^**a**^
	Surgery-related anxiety	7.29 ± 2.28	7.06 ± 1.91	0.75 ^**a**^
	Information desire component	7.17 ± 1.74	6.71 ± 1.99	0.47 ^**a**^
	Combined anxiety component	13.71 ± 4.55	13.41 ± 4.02	0.85 ^**a**^
QOR (Mean ± SD)	Total QOR	159.9 ± 13.93	135.6 ± 8.43	**0.0001** ^**a**^
	QOR-Physical	48.13 ± 4.73	39.81 ± 3.29	**0.0001** ^**a**^
	QOR- Emotional	34 ± 2.99	28.25 ± 3.11	**0.0001** ^**a**^
	QOR- Psychological	33.69 ± 3.98	28.44 ± 4.08	**0.0014** ^**a**^
	QOR-Physical Independence	13.94 ± 4.18	11.75 ± 3.11	**0.038** ^**a**^

PTG group: the patient group that received placebo

MTG group: the patient group that received melatonin 60 mg

APAIS: The Amsterdam Preoperative Anxiety and Information Scale; MMAS: Morisky Medication Adherence Scale; MTG: Melatonin Treated group; PTG: Placebo-treated Group; QOR: Quality of Recovery; VAS: Visual Analogue Scale for Anxiety

^a^ Statistical test: Unpaired t-test; Statistical significance (*P* < 0.05)

### End of study evaluation

#### Melatonin effect on apoptosis, inflammatory and cardiac biomarkers

**Fig. 2:**
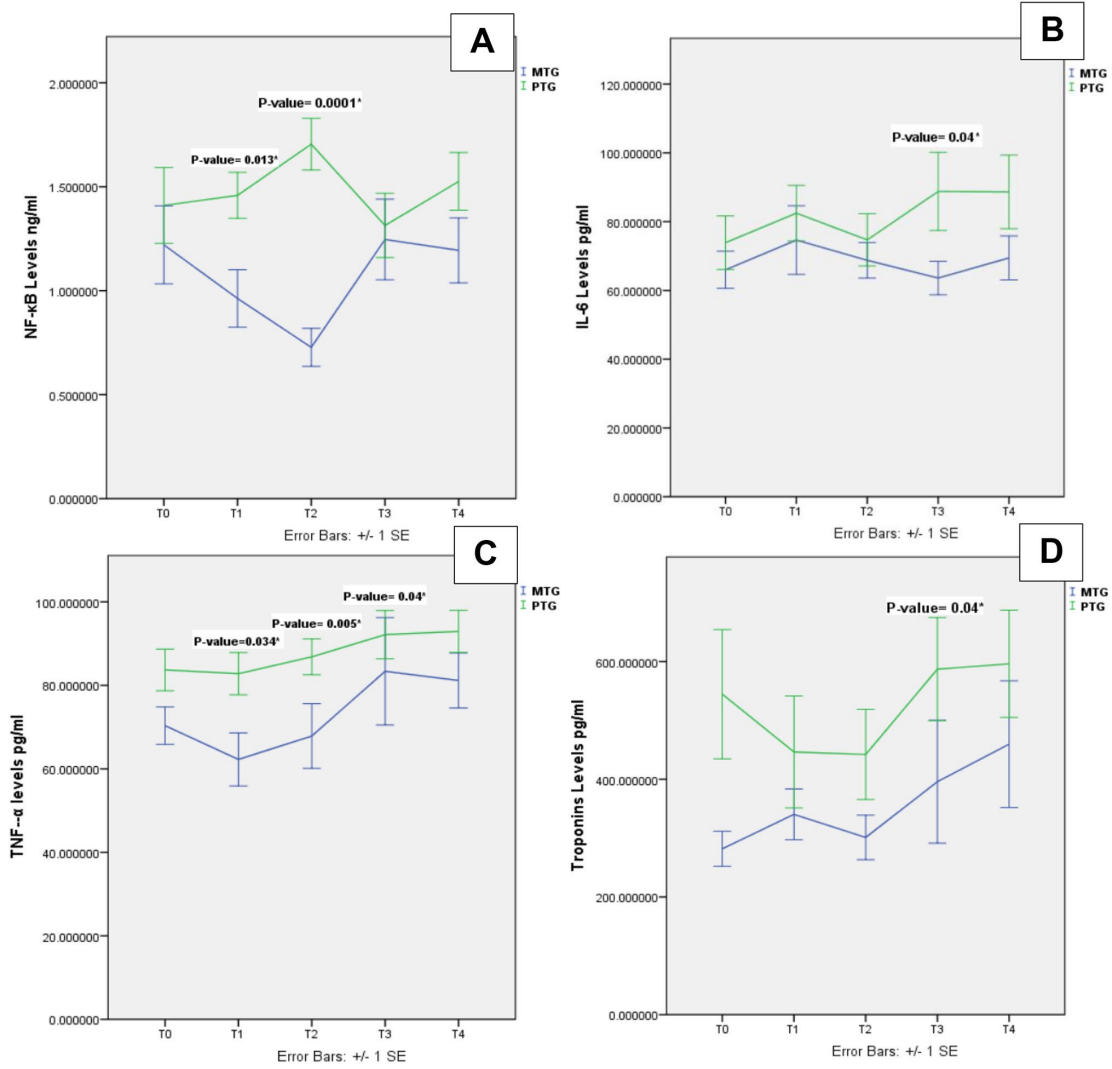
Nuclear factor Kappa Beta (NF-κB), interleukin 6 (IL-6), tumor necrosis factor (TNF-α) and Troponins levels in the plasma of the participants in the Melatonin-treated group (MTG) and placebo-treated group(PTG). **A**: NF-κB protein expression levels in both groups. **B**: IL-6 protein expression levels in both groups. **C**: TNF-α protein expression levels in both groups. **D**: Cardiac Troponins I protein expression levels in both groups. T0, baseline; T1, pre-cross clamp; T2, after cross clamp removal; T3, 6 h after cross-clamp removal; t4T4, 24 h after cross-clamp removal. *P < 0.05

NF-κB levels declined at T1 (*p* = 0.013) and T2 (*p* = 0.0001) in MTG compared to PTG (Fig. 2A**).** Regarding the change over time, there was a statistically significant change in the MTG (*p* = 0.007). Post-hoc analysis showed a statistically significant difference between T2 and T3 (adjusted *p* = 0.017), and T2 and T4 (adjusted *p* = 0.017) in the MTG. Amongst PTG, a statistically significant difference was found between T2 and T3 (adjusted *p* = 0.04).

TNF-α levels declined in MTG at T1(*p* = 0.034) versus PTG. At T2(*p* = 0.005), and T3(*p* = 0.04), TNF-α significantly increased in PTG versus MTG. (Fig. 2C). In the MTG, there was a statistically significant change over time (*p* = 0.035). Post-hoc analysis showed a statistically significant difference between T1 and T4 (adjusted *p* = 0.04). However, regarding PTG, there was no statistically significant difference over time.

Troponins significantly increased in PTG at T3 (*p* = 0.04) versus MTG (Fig. 2D). In the MTG, the troponin levels were maintained amongst all the measured time points (T; 0, 1, 2, 3 and 4), while a significant increase occurred at T3 in the PTG. However, there was no statistically significant difference was observed over time in MTG or PTG at all other time points.

Like troponins, IL-6 significantly increased in PTG versus MTG at T3 (*p* = 0.04) (Fig. 2B), with no statistically significant difference over time in either the MTG or the PTG.

#### Melatonin effect on operative and intensive care unit parameters

There were no significant differences in the operative and intensive care unit parameters between the two groups except for the intubation time (Table 6). The melatonin-treated group had a statistically significant shorter intubation time than did the placebo group (*p* = 0.03).

**Table 6:** Operative and Intensive Care Unit data in both groups

Variable	MTG group (*n* = 17)	PTG group (*n* = 17)	*P*-value
No. of arteries repaired (Median ± IQR)	3 (2–3)	3 (3–4)	0.18 ^**a**^
Surgery duration (minutes) (Median ± IQR)	360 (360–420)	360 (360–425)	0.62^**a**^
CBP duration (minutes) (Mean ± SD)	156.7 ± 42.2	158.8 ± 38.11	0.88 ^**b**^
CCT (minutes) (Mean ± SD)	97.82 ± 32.04	93.12 ± 24.48	0.63 ^**b**^
Intubation time (hours) (Median ± IQR)	15 (12–20)	18 (15–25)	**0.03** ^**a**^
Intensive Care Unit Length of Stay (days) (Median ± IQR)	3 (2-3.5)	3 (2–4)	0.71^**a**^
Hospital Length of Stay (days) (Mean ± SD)	7.94 ± 2.75	7.64 ± 2.52	0.75 ^**a**^

PTG group: the patient group that received placebo

MTG group: the patient group that received melatonin 60 mg

CBP: Cardiopulmonary Bypass; CCT: Cross Clamp Time; MTG: Melatonin Treated Group; PTG: Placebo-treated group

^a^ Statistical test: Mann-Whitney test

^b^ Statistical test: Unpaired t-test

Statistical significance (*P* < 0.05)

#### Melatonin effect on clinical outcomes

Heart rate was maintained from baseline to after 24 h in the MTG group, while there was a significant increase in the PTG. However, there were no statistically significant differences between the groups at baseline or 24 h post-operative (Table 3).

The oxygen saturation and systolic blood pressure were comparable between the 2 groups over time. However, Friedman’s test revealed a significant decrease in diastolic blood pressure in the MTG over time. Post-hoc tests revealed differences between baseline and 24 h postoperative. On the contrary, PTG showed no significant change over time. There was a statistically significant difference between the two groups at 24 h post-operative (Table 3).

#### Melatonin effect on ICU and hospital length of stay

Both the overall ICU and hospital length of stay were not significantly different between the 2 groups. However, the MTG showed a 3.78% reduction in hospital stay and a 5.7% reduction in ICU stay compared with the PTG. There was no significant difference between the two groups regarding post-CABG complications (Table 7).

**Table 7:** Post coronary artery bypass grafting complications in both groups

Variable		MTG group (*n* = 17)	PTG group (*n* = 17)	*P*-value
Atrial Fibrillation	Yes	1	2	1 ^**a**^
	No	16	15	
Ventricular Fibrillation	Yes	1	1	1 ^**a**^
	No	16	16	
Asystole	Yes	2	3	1 ^**a**^
	No	15	14	
Wound infection	Yes	2	1	1 ^**a**^
	No	15	16	
In-hospital Death	Yes	1	3	0.6 ^**a**^
	No	16	14	

PTG group: the patient group that received placebo

MTG group: the patient group that received melatonin 60 mg

MTG: Melatonin Treated Group; PTG: Placebo-treated group

^a^ Statistical test: Fisher’s Exact test

Statistical significance (*P* < 0.05)

#### Melatonin effect on medication adherence and quality of recovery

Regarding medication adherence and the quality of recovery questionnaire, the scores of the medication adherence questionnaire were comparable between the two groups. However, the MTG had significantly lower scores for the individual domains of the Quality of Recovery Questionnaire versus the PTG as well as the total scores (Table 5).

#### Melatonin effect on laboratory parameters

Laboratory parameters, except for blood glucose, were not significantly different 24 h after cross-clamp removal between the two groups. There was a significant difference between the 2 groups in the 24 post cross-clamp glucose levels as well as the percent change between baseline and 24 h. Blood glucose levels significantly decreased in the MTG group but significantly increased in the PTG from baseline to 24 h. post clamp removal (Table 4).

#### Melatonin safety

##### Melatonin safety

Melatonin 60 mg was well-tolerated without any reported side effects.

#### Correlation analysis

A correlation analysis between the quality of the recovery questionnaire total score and TNF- α showed a statistically significant negative correlation at T0 (R^2^ = 0.497, p-value = 0.0001). Additionally, a statistically significant negative correlation between the total score of the quality of recovery questionnaire and NF-κB was observed at T2 (R^2^ = 0.247, p-value = 0.003). Furthermore, the quality of recovery questionnaire total score and IL-6 showed a statistically significant negative correlation at T3 (R^2^ = 0.142, p-value = 0.028). Regarding the intubation time, it showed a statistically significant positive correlation with TNF- α at T2 (R^2^ = 0.253, p-value = 0.002), troponins at T2 (R^2^ = 0.166, p-value = 0.017), and IL-6 at T1 (R^2^ = 0.125, p-value = 0.04) and T2 (R^2^ = 0.12, p-value = 0.046).

## Discussion

Currently, there is no effective treatment for ischemia/reperfusion injury, a devastating consequence of CABG surgery. In search of innovative therapies, melatonin is a potentially promising candidate with various effects that could ameliorate the challenging IR insults. In the present study, melatonin administration resulted in statistically significant differences in the levels of NF-kB, IL6, TNF-α, and troponin-I versus placebo in favor of the MTG group. Additionally, melatonin decreased DBP 24 h post-operative, reduced intubation time, and improved the quality of recovery compared to placebo.

Some CABG modalities such as the off-pump technique avoid some complications of cardiopulmonary bypass [26]. Besides, the minimized closed circuit CABG technique has been shown to decrease the inflammatory response [27]. Ischemia/reperfusion injury occurs due to oxidative stress and activation of inflammatory pathways during conventional CABG surgery [13]. This myocardial injury can lead to postoperative morbidity and heart failure in the long run [28]. Therefore, approaches aimed at modulating ischemia/reperfusion have been recognized as valuable approaches for preventing postoperative complications and mortality associated with CABG.

Studies have shown that melatonin can play a protective role in I/R injury, by alleviating inflammation, and oxidative stress [29–31]. However, despite the antioxidant and anti-inflammatory effects of melatonin, only a few studies have demonstrated its protective effect against myocardial ischemia/reperfusion injury and inflammation [4, 21–24].

The NF-κB signaling pathway is essential for the regulation of immunity and inflammation [32]. The excessive inflammatory response during ischemia/reperfusion is mediated by the activation of the NF-κB pathway [33]. Activated NF-kB upregulates the production of proinflammatory cytokines such as IL-1, IL-6, TNF- and CRP [34].

We chose NF-κB, TNF-α, and IL-6 as monitoring factors; these factors reflect the levels of inflammation and apoptosis in the body. Both inflammation and apoptosis play vital roles in the incidence of the devastating effects of ischemia/reperfusion injury in CABG surgery. To our knowledge, the current study is the first to investigate the effect of high-dose melatonin (60 mg) on NF-κB levels in CABG patients. Our study has ClinicalTrials.gov registration number: NCT05552586.

At baseline, both groups exhibited comparable levels of NF-κB, TNF-α, troponins, and Il-6.

Our results revealed a significant decline in NF-κB levels in the MTG compared to the PTG with a significant decline only in the MTG over time. Zhao et al., investigated the protective effect of melatonin on brain ischemia and reperfusion after the administration of 6 mg/d melatonin orally from 3 days before surgery to 3 days after carotid endarterectomy surgery. Compared with those in the placebo group, the levels of NF-κB in the melatonin-treated group were statistically significantly lower [31]. This result is consistent with our findings.

The decrease in the expression of NF-κB induced by pretreatment with melatonin, as reported in the present study, might have contributed to the inhibition of the upregulation of downstream inflammatory molecules.

Accordingly, TNF-α levels declined significantly in the MTG relative to the PTG. Regarding the change over time, there was a statistically significant decline in the level of TNF-α in the MTG versus the PTG that showed no change. A study by Dwaich et al. showed similar results in CABG patients. This study was performed to examine the protective role of melatonin in ameliorating the degree of myocardial injury in patients undergoing CABG, and to determine whether this effect is dose related. Both groups showed a significant decrease in TNF-α levels compared to those in the placebo group [23]. Similarly, in the study by Zhao et al., the melatonin-treated group showed a statistically significant decrease in the level of TNF-α compared to the placebo group [31]. That finding was in agreement with our findings.

In the present study, there was a statistically significant difference in the troponin I levels between both groups at T3. Troponin levels were maintained among all the measured time points (T; 0, 1, 2, 3 and 4) in the MTG, while an increase occurred at T3 in the PTG. However, troponins didn’t show any statistically significant change over time in both groups. This could be due to the cardioprotective effect of cardioplegia during CABG. Similarly, Dwaich et al. reported a decrease in troponin levels in melatonin groups compared with the placebo group [35].

Despite sampling at different time points, the results of the study by Shafiei et al. were in agreement with our findings and melatonin succeeded in decreasing the levels of troponin and TNF-α in patients undergoing coronary artery bypass grafting surgery [21].

Finally, the IL-6 level was statistically significantly lower in MTG than in the PTG. However, IL-6 didn’t show any statistically significant change over time in both groups.

Similarly, Zhao et al., showed a statistically significant decrease in the level of IL-6 in patients who underwent carotid endarterectomy in the melatonin group compared to the placebo group.

Collectively, all of the aforementioned findings highlight the effective role of melatonin in halting the devastating effects of IR injury by modulating the inflammatory and apoptotic markers in patients undergoing CABG. This could help improve the injury resulting from exposure to ischemia and sudden reperfusion and potentially exert a cardioprotective effect and minimize the postoperative complications that might occur.

This was also apparent in the current study for various clinical parameters and outcomes. In terms of the vital parameters, the DBP significantly decreased in the MTG compared to the PTG, with a significant decline occurring only in the MTG over time. This finding was in agreement with the study results of Raygan et al., who showed a statistically significant decrease in DBP after taking 10 mg/day melatonin for 12 weeks, compared with placebo in diabetic patients with CHD [36].

Additionally, the use of melatonin was accompanied by a decrease in the intubation time compared to that of the placebo group (*p* = 0.0315). Moreover, despite not reaching significance, there was a 3.78% reduction in hospital stay from 7.94 days in PTG to 7.64 days in the MTG and a 5.7% reduction in ICU stay from 2.971 in PTG to 2.8 days in the MTG.

Javaherforoosh et al. investigated the effect of 3 mg of melatonin administered the evening before the operation, the morning of surgery, and the second postoperative day in CABG patients [37]. In agreement with our results, the melatonin group showed a statistically significant decrease in mechanical ventilation duration compared to the control group [37]. However, the decrease in ICU length of stay was in contrast to our findings. This discrepancy in results may be due to the difference in dosing regimens between the two studies.

The previously mentioned effects of melatonin on inflammation and apoptosis may explain its positive impact on the clinical outcomes of these patients. Great attention has been given to the cardioprotective function of melatonin [38–40].

Preoperative anxiety can contribute to negative patient outcomes. In the present study, we also evaluated the impact of melatonin on preoperative anxiety. Regarding the level of preoperative anxiety, there was no statistically significant difference between both groups regarding the APAIS score or VAS score.

Nevertheless, the total score and the individual score of each domain of the quality of recovery questionnaire were significantly improved in MTG compared to PTG. This could be attributed to melatonin’s hypnotic and sedative effects.

Similarly, Michal et al. evaluated the effect of 5 mg melatonin premedication on postoperative recovery in patients undergoing bariatric surgery [41]. The use of melatonin premedication improved the quality of recovery 1 day after bariatric surgery as measured by the QoR-15, specifically the quality of sleep and pain levels.

This study was the first to investigate the correlation between the quality of recovery and different markers such as NF-κB, TNF- α, and IL-6. This study also showed a significant negative correlation between the total score of quality of recovery questionnaire and TNF- α at T0 (*r*= -0.705, p-value = 0.0001), NF-κB at T2 (*r*=-0.487, p-value = 0.003) and IL-6 at T3 (*r*= -0.377, p-value = 0.028), indicating the contribution of halting these inflammatory mediators and improving the quality of life by melatonin administration.

The intubation time, it showed statistically significant positive correlations with TNF- α at T2 (*r* = 0.503, p-value = 0.002), troponins at T2 (*r* = 0.407, p-value = 0.017), and IL-6 at T1 (*r* = 0.353, p-value = 0.04) and T2 (*r* = 0.344, p-value = 0.046), also highlighting their impact of reducing the levels of proinflammatory markers and its impact on relation to patient recovery.

Despite the notable variation in the dosing regimens and durations used in the previous studies, no studies have reported toxicity. Even for very high doses of melatonin (300 mg/kg) used by Nickkholgh et al., in their pilot study in 2011, or by Galley et al., in 2014, no adverse effects related to the treatment were reported [25, 42].This finding supports the excellent safety profile of melatonin, which has also been documented in the literature. Based on the results and recommendations of the study by Zhao et al., and based on the excellent safety profile of melatonin, the current study used a higher dose (60 mg) than those previously reported [31].

Last, melatonin administration resulted in a significant control and decrease in blood glucose levels compared to PTG which showed an increase in blood glucose over time, highlighting its beneficial role in blood glucose control. In line with our results, Raygan et al., showed that taking 10 mg/day melatonin for 12 weeks, compared with the placebo, significantly reduced fasting plasma glucose in diabetic patients with CHD [36].

No statistically significant difference was detected between both groups regarding postoperative complications. Melatonin 60 mg was well-tolerated with no reported undesired side effects from its use. In the study by Mansilla-Roselló 60 mg of melatonin didn’t cause any side effects in surgical patients with severe sepsis [43]. All of these findings confirm the results of the current study, which showed an excellent safety profile of melatonin. In contrast, Zoe et al. reported that high dose melatonin (> 10 mg) did not cause a detectable increase in serious adverse events or withdrawal due to adverse events, but did increase the risk of adverse events such as drowsiness, headache, and dizziness [44], which was not observed in the current study.

This study has several strengths. First, this is the first study to use such a high dose in CABG patients. Second, no other studies have evaluated the effect of melatonin on the NF-KB parameter in CABG patients. Third, the current study demonstrated that melatonin might protect against myocardial I/R injury in CABG patients via various effects on ameliorating inflammation and apoptosis and improving clinical outcomes.

However, our study has several limitations. First, the dose-dependent effects of melatonin still need to be evaluated to further assess the protective effects observed in this study. Second, the relatively short duration of preoperative melatonin intake is another drawback. Third, melatonin levels weren’t measured in this study. Finally, the sample size should be expanded in future studies.

In summary, this study revealed that [1] CABG can increase the levels of apoptotic and inflammatory markers, and that (ii) melatonin can ameliorate the ischemia/reperfusion injury by decreasing the level of NF-κB, a key regulator of the inflammatory process, as reflected by the decrease in the levels of inflammatory markers including IL-6 and TNF-α,

(iii) Clinically, melatonin significantly decreased DBP, intubation time, and improved the quality of recovery. Additionally, hospital and ICU lengths of stay were decreased in MTG, however, they weren’t statistically significant.

Based on the results of this study, we believe that melatonin may be a potential preventive therapy for post-CABG myocardial I/R injury.

## Conclusion

Melatonin showed its protective effects against myocardial I/R injury in CABG patients. It significantly decreased the levels of NF-κB, a key regulator of the inflammatory process, and this effect was reflected by the decrease in the levels of inflammatory markers including IL-6 and TNF-α. In addition, melatonin successfully decreased the intubation time and the quality of recovery on postoperative day 4. Additionally, melatonin was well-tolerated with no reported undesired side effects from its use. Larger clinical trials are required to confirm the beneficial effects of melatonin on myocardial I/R injury in CABG patients.

### Patients and methods

#### Study design

The was a prospective, randomized; single-blinded placebo-controlled study conducted at the cardiothoracic surgery department, of the Academy of Cardiovascular and Thoracic Surgery at Ain Shams University Hospital.

#### Ethical consideration

The study was conducted according to the Good Clinical Practice guidelines and followed the ethical principles of the Declaration of Helsinki (as revised in 2013). This study also applied the CONSORT guidelines and the ICMJE recommendations. The protocol was revised and approved by the ethics committee for experimental and clinical studies at the Faculty of Pharmacy and Faculty of Medicine, Ain Shams University, Cairo, Egypt. The ClinicalTrials.gov registration number is: NCT05552586. All participants were given information sheets explaining all the details pertaining to the study. Informed consent was obtained from all patients before their inclusion in the study.

#### Patients and treatment

The study included all patients who were to undergo an elective on-pump CABG, agreed to participate in the study and could be reached by phone.

The exclusion criteria included the following: urgent CABG surgery with no adequate time to receive the study drug preoperatively for 5 days, reoperative (CABG), off-pump CABG, concomitant renal failure, severe respiratory problems, previous stroke or significant cerebrovascular disease, hepatitis B or C, HIV, inflammatory diseases (ex. Rheumatoid arthritis, psoriasis, multiple sclerosis, etc.), an ejection fraction (EF%) lower than 40%, CABG surgery with simultaneous heart valve repair or replacement, resection of a ventricular aneurysm, or other operations, pregnancy, and lactation.

Eligible patients were randomly allocated via stratified randomization into either the melatonin-treated group (MTG) coded as A or the placebo-treated group (PTG) coded as B. Thirty-four patients were coded as 1–34. Patient allocation into either group was performed using the 4-sized block randomization method by the principal investigator. The possible sequences were as follows: ABBA, AABB, BABA, BBAA, BABA and BAAB. The list of random numbers was generated by randomizer.org.

Melatonin-treated Group (MTG) (*n* = 17) patients received 60 mg/day melatonin capsules orally once daily starting from day five before surgery in addition to the standard of care. Placebo-treated group (PTG) (*n* = 17) patients received placebo with the same appearance and packaging and the same regimen for five days before surgery in addition to the standard of care.

All participants received standard-of-care treatment according to the clinical guidelines. Allocation to either group didn’t affect the quality of care received by the patients.

All patients were offered comprehensive health care services provided by the clinical pharmacist plus standard care provided by the physician.

Melatonin (Melatonin Max^®^) was obtained from the manufacturer “Perfect Vitamin Products, Carson City, NV, USA’’. The placebo was manufactured and supplied by the “Memphis Company for Pharmaceutical and Chemical Industries”, Cairo, Egypt.

Treatment intake was ensured by drug or placebo administration at the cardiothoracic surgery department. The drug or placebo was supplied by a designated nurse at the unit, every night for 5 days before surgery.

### Methods

The following data were collected for both groups:

#### Patients’ demographics, social, medical and medication history

1. The demographics included age, sex, weight, height, body mass index [BMI], and waist circumference.
2. Social history: marital status, education, occupation, smoking status, lifestyle habits.
3. Medical history: duration of CHD, number of admissions, comorbidities and past and concurrent diseases.
4. Medication history: Past and concurrent prescribed medications and OTC medications.

#### Evaluation of risk factors

Hypertension, dyslipidemia, diabetes mellitus, family history of ischemic heart disease, Smoking (current smoker, nonsmoker, or ex-smoker), obesity (BMI ≥ 30) / overweight (BMI [25–30]) and lifestyle habits (exercise and diet) were recorded.

#### Preoperative anxiety

It was assessed using the Visual Analogue Scale for Anxiety (VAS-A), a visual analog numerical scale ranging from 0 (no anxiety) to 100 (worst possible anxiety). Additionally, the Amsterdam Preoperative Anxiety and Information Scale (APAIS), a validated instrument used to assess preoperative anxiety, was used and included 6 questions with scores between 1(none) and 5 (most) for each answer, with 30 points in total. The APAIS has an anxiety component (APAISa,4 questions, maximum 20 points) and a knowledge component (APAISk,2 questions, maximum 10 points).

#### Medication 

It was assessed using the Morisky Medication Adherence Scale (MMAS).

#### Clinical evaluation

All patients were subjected to a continuous assessment of vital signs (blood pressure, heart rate and blood oxygen saturation level (SPo2), together with electrocardiography (ECG) and echocardiographic evaluation before and after 1 day of the operation.

#### Laboratory evaluation

**Routine biochemical parameters** including fasting blood glucose, total cholesterol, triglycerides, high-density lipoprotein, low-density lipoprotein cholesterol, serum urea, creatinine, sodium, potassium, complete blood count and prothrombin time (PT), all were measured preoperatively and 1 day postoperatively.
The following **viral screening tests** were also performed before surgery: anti-hepatitis C virus antibody (anti-HCV Ab), hepatitis B surface antigen (HBs Ag), and human immunodeficiency virus (HIV).
**3. Blood Biomarkers**: The assessed biomarkers for all patients were plasma NF-κB as the primary outcome and tumor necrosis factor (TNF-α), cardiac troponin I, and IL-6 levels as the secondary outcomes.

Serial arterial blood samples were collected from all patients as follows:

Blood samples (4 ml) were collected before melatonin or placebo administration before the aortic cross-clamp was placed, after the aortic cross-clamp was released, and 6 and 24 h after the operation. Blood was placed in a test tube containing anticoagulant (disodium EDTA (22 mg/ml)), mixed thoroughly, and centrifuged at 3000 rpm for 15 min. The plasma was kept frozen at − 80 °C.

The levels of biochemical markers (IL-6, TNF-α, cardiac troponins I, and NF-κb) were measured with enzyme-linked immunosorbent assay (ELISA) kits. Commercial ELISA kits “Bioassay Technology Laboratory™” (Cat. No. E0090Hu, E0082Hu, E1242Hu, and E0690Hu) were used to measure IL-6, TNF- α, cardiac troponin I, and NF-κB. ELISA procedures were performed according to the manufacturer’s instructions. The samples were then measured at an absorbance of 450 nm, and the mean concentrations were calculated (Bioassay Technology Laboratory, Shanghai Crystal Day Biotech Co., Ltd., Shanghai, China).

#### The ICU and hospital length of stay

Were recorded for all patients

#### Quality of recovery

Five general quality-of-life dimensions were measured with the QoR-40 score: physical comfort (12 items), emotional state (9 items), physical independence (5 items), psychological support (7 items), and pain (7 items). Each item was graded on a 5-point Likert scale, and the global scores ranged from 40 (extremely poor QoR) to 200 (excellent QoR). The QoR-40 form was provided to patients for completion on postoperative day 4.

#### Safety assessment

Safety issues: Patients in the MTG were followed up via phone if they had any complaints of melatonin administration 5 days before CABG.

#### Surgical procedure

All the CABG surgeries performed in the current study were performed by the same 2 surgeons with the assistance of the surgical team of the cardiothoracic surgery department, Academy of Cardiovascular and Thoracic Surgery using the on-pump coronary artery bypass grafting surgical technique. All the surgeries were scheduled and performed in the morning starting at 9 am. Surgeons and patients were blinded to treatment. LivaNova Sorin Stockert S5 with a console for four roller pumps and 1 twin pump was the heart lung machine used in all study patients. Regarding the oxygenator, Inspire oxygenator (Sorin) 8 with integrated arterial filter was used. Heparin was dosed at 300 IU/kg intravenous. Activated clotting time was checked 3 min after heparin administration. When ACT was less than 400 s, another dose of 500 IU was given, and ACT was repeated to ensure ACT < 400 s. Then ACT was repeated every half an hour during the operation till the patient was off bypass from the heart-lung machine. Clinical chemistry analyzer in the operation room was used to perform faster laboratory testing including: blood gases (pO2, pCO2), pH, electrolytes (sodium, potassium, calcium), Hematocrit, hemoglobin, blood glucose and ACT testing. Post-operative complications: respiratory failure, renal failure, AF, sternal wound infection, VTE, stroke, hemorrhage, and cardiac arrest were assessed.

#### Sample size

By using the G program sample size calculation, setting the power at 80% and the α error at 5%, and after reviewing previous study results by Zhao et al. [29], we used nuclear factor kappa-b (NF-KB) as a key variable and primary outcome. Levels of NF-kB were measured at 6 h of carotid endarterectomy (∆ mean = 213 pg/ml, ∆SD = 37pg/ml). A sample of 12 patients in each group was required. Accounting for a drop out of at least 25%, 34 patients have been enrolled in the study (17 patients on the MTG, and 17 patients on the PTG).

#### Statistical analysis

Statistical analyses were performed using the IBM Statistical Package for Social Statistical Analyses version 21 computer software (SPSS Inc. which was acquired from IBM in 2009, Chicago, USA). The Data are expressed as the median and interquartile range for quantitative nonparametric measurements, as the mean and standard deviation for parametric data, and as numbers and percentages for categorical data. Fisher’s exact test and chi-square test were used to compare categorical data. The Friedman test was used for within-group comparisons. The percent change after treatment between the two groups was compared using an unpaired t-test and Mann-Whitney U test. Changes in apoptotic and inflammatory marker levels at different time points were compared using the Mann-Whitney U test using Bonferroni adjustment. Spearman’s correlation test was performed to assess the correlation between the total score of the quality of recovery questionnaire and NF-kB, TNF-α, troponins and IL-6 levels. Additionally, correlations between intubation time and NF-kB, TNF-α, troponins and IL-6 levels were investigated using the same test. P-value < 0.05 was considered to be significant.

## Data Availability

No datasets were generated or analysed during the current study.

## References

[1] BHF (2023) British Heart Foundation Factsheet. https://www.bhforguk/-/media/files/for-professionals/research/heart-statistics/bhf-cvd-statistics-global-factsheetpdf

[2] Raparelli V, Nocella C, Proietti M et al (2022) Testosterone-to-estradiol ratio and platelet thromboxane release in ischemic heart disease: the EVA project. J Endocrinol Investig :1–11 10.1007/s40618-022-01771-0 PMC9184432 35262860

[3] Bond CJ, Milojevic M, He C et al (2021) Quality Improvement: arterial grafting redux, 2010:2019. Ann Thorac Surg 112:22–30 33189668 10.1016/j.athoracsur.2020.08.072

[4] Farshidianfar M, Ardekani A, Tabrizi R et al (2023) Effects of Melatonin on Cardiac Injury and inflammatory biomarkers in patients undergoing coronary artery bypass graft surgery: a Meta-analysis. Cardiol Therapy 12:11–20 10.1007/s40119-022-00287-1 PMC9986370 36352301

[5] Nasseh N, Khezri MB, Farzam S, Shiravandi S, Shafikhani AA (2022) The effect of melatonin on cardiac biomarkers after coronary artery bypass graft surgery: a double-blind, randomized pilot study. J Cardiothorac Vasc Anesth 36:3800–3805 35817673 10.1053/j.jvca.2022.06.003

[6] Zhou H, Li D, Zhu P et al (2017) Melatonin suppresses platelet activation and function against cardiac ischemia/reperfusion injury via PPARγ/FUNDC1/mitophagy pathways. J Pineal Res 63:e12438 10.1111/jpi.12438 28749565

[7] Amini-Farsani Z, Yadollahi-Farsani M, Arab S, Forouzanfar F, Yadollahi M, Asgharzade S (2021) Prediction and analysis of microRNAs involved in COVID-19 inflammatory processes associated with the NF-kB and JAK/STAT signaling pathways. Int Immunopharmacol 100:108071 34482267 10.1016/j.intimp.2021.108071 PMC8378592

[8] Izzo C, Vitillo P, Di Pietro P et al (2021) The role of oxidative stress in Cardiovascular Aging and Cardiovascular diseases. Life 11:60 33467601 10.3390/life11010060 PMC7829951

[9] Wang X, Han W, Zhang Y, et al. (2022) Soluble Epoxide Hydrolase Inhibitor t-AUCB Ameliorates Vascular Endothelial Dysfunction by Influencing the NF-&kappa;B/miR-155-5p/eNOS/NO/I&kappa;B Cycle in Hypertensive Rats. Antioxidants 11:1372. 10.3390/antiox11071372 PMC9311992 35883863

[10] Ji C, Pan Y, Xu S et al (2021) Propolis ameliorates restenosis in hypercholesterolemia rabbits with carotid balloon injury by inhibiting lipid accumulation, oxidative stress, and TLR4/NF-κB pathway. J Food Biochem 45:e13577 33729587 10.1111/jfbc.13577

[11] Ding X, Zhu C, Wang W, Li M, Ma C, Gao B (2023) SIRT1 is a regulator of autophagy: implications for the progression and treatment of myocardial ischemia-reperfusion. Pharmacol Res :106957 10.1016/j.phrs.2023.106957 37820856

[12] Gunata M, Parlakpinar H (2021) A review of myocardial ischaemia/reperfusion injury: pathophysiology, experimental models, biomarkers, genetics and pharmacological treatment. Cell Biochem Funct 39:190–217 32892450 10.1002/cbf.3587

[13] Bermudez-Gonzalez JL, Sanchez-Quintero D, Proaño-Bernal L et al (2022) Role of the antioxidant activity of Melatonin in Myocardial Ischemia-Reperfusion Injury. Antioxidants 11:627 35453312 10.3390/antiox11040627 PMC9032762

[14] Wang L, Wang W, Han R, Liu Y, Wu B, Luo J (2023) Protective effects of melatonin on myocardial microvascular endothelial cell injury under hypertensive state by regulating Mst1. BMC Cardiovasc Disord 23:179 37005605 10.1186/s12872-023-03159-1 PMC10068162

[15] Zhou H, Ma Q, Zhu P, Ren J, Reiter RJ, Chen Y (2018) Protective role of melatonin in cardiac ischemia-reperfusion injury: from pathogenesis to targeted therapy. J Pineal Res 64:e12471 10.1111/jpi.12471 29363153

[16] Misaka T, Yoshihisa A, Yokokawa T et al (2019) Plasma levels of melatonin in dilated cardiomyopathy. J Pineal Res 66:e12564 30715754 10.1111/jpi.12564 PMC6593840

[17] Poeggeler B, Reiter RJ, Tan DX, Chen LD, Manchester LC (1993) Melatonin, hydroxyl radical-mediated oxidative damage, and aging: a hypothesis. J Pineal Res 14:151–168 8102180 10.1111/j.1600-079x.1993.tb00498.x

[18] Lochner A, Marais E, Huisamen B (2018) Melatonin and cardioprotection against ischaemia/reperfusion injury: what’s new? A review. J Pineal Res 65:e12490 29570845 10.1111/jpi.12490

[19] Sakotnik A, Liebmann P, Stoschitzky K et al (1999) Decreased melatonin synthesis in patients with coronary artery disease. Eur Heart J 20:1314–1317 10462465 10.1053/euhj.1999.1527

[20] Shafiq MH, Jabeen M, Shakeel I, Zaidi E (2023) Letter to editor: melatonin as a Cardio-Protectant: postoperative insights. Curr Probl Cardiol 49:102142 37863461 10.1016/j.cpcardiol.2023.102142

[21] Shafiei E, Bahtoei M, Raj P et al (2018) Effects of N-acetyl cysteine and melatonin on early reperfusion injury in patients undergoing coronary artery bypass grafting: a randomized, open-labeled, placebo-controlled trial. Medicine 97 10.1097/MD.0000000000011383 PMC6078764 30045259

[22] Hajhossein-Talasaz A, Dianatkhah M, Ghaeli P, Salehiomran A, Dianatkhah M (2022) Possible effects of melatonin on reperfusion injury following coronary artery bypass graft surgery. ARYA Atherosclerosis 18:1 36819839 10.48305/arya.v18i0.2208 PMC9931614

[23] Dwaich KH, Al-Amran FG, Al-Sheibani BI, Yousif NG, Hadi NR (2018) The cardioprotective role of melatonin against myocardial injury in patients undergoing coronary artery bypass grafting surgery. Vascular Invest Therapy 1:41–49

[24] Barati S, Jahangirifard A, Ahmadi ZH, Tavakoli-Ardakani M, Dastan F (2021) The effects of melatonin on the oxidative stress and duration of atrial fibrillation after coronary artery bypass graft surgery: a randomized controlled trial. Endocrine, metabolic & Immune disorders-drug targets (formerly current drug targets-Immune. Endocr Metabolic Disorders) 21:1142–1149 10.2174/1871530320666200728152307 32723264

[25] Nickkholgh A, Schneider H, Sobirey M et al (2011) The use of high-dose melatonin in liver resection is safe: first clinical experience. J Pineal Res 50:381–388 21480979 10.1111/j.1600-079X.2011.00854.x

[26] Zubarevich A, Kadyraliev B, Arutyunyan V, Chragyan V, Askadinov M, Sozkov A et al (2020) Tsagakis K. On-pump versus off-pump coronary artery bypass surgery for multi-vessel coronary revascularization. J Thoracic Dis 12(10):5639 10.21037/jtd-20-1284 PMC7656392 33209396

[27] Albacker TB, Fouda M, Bakir BM, Eldemerdash A (2021) The effect of using the minimized cardio-pulmonary bypass systems for coronary artery bypass grafting in diabetic patients. J Cardiothorac Surg 16:1–7 10.1186/s13019-021-01551-6 PMC8182931 34099011

[28] Skeffington KL, Moscarelli M, Abdul-Ghani S et al (2022) Pathology-related changes in cardiac energy metabolites, inflammatory response and reperfusion injury following cardioplegic arrest in patients undergoing open-heart surgery. Front Cardiovasc Med 9 10.3389/fcvm.2022.911557 PMC9354251 35935655

[29] Dun R-l, Lan T-y, Tsai J et al (2022) Protective effect of melatonin for renal ischemia-reperfusion injury: a systematic review and meta-analysis. Front Physiol 12:791036 35095558 10.3389/fphys.2021.791036 PMC8793910

[30] Zhang F, Lin B, Huang S et al (2023) Melatonin alleviates retinal ischemia–reperfusion Injury by inhibiting p53–Mediated ferroptosis. Antioxidants 12:1173 37371903 10.3390/antiox12061173 PMC10295547

[31] Zhao Z, Lu C, Li T et al (2018) The protective effect of melatonin on brain ischemia and reperfusion in rats and humans: in vivo assessment and a randomized controlled trial. J Pineal Res 65:e12521 30098076 10.1111/jpi.12521

[32] Casper E (2023) The crosstalk between Nrf2 and NF-κB pathways in coronary artery disease: can it be regulated by. SIRT6? Life Sci 330:122007 37544377 10.1016/j.lfs.2023.122007

[33] Algoet M, Janssens S, Himmelreich U et al (2023) Myocardial ischemia-reperfusion injury and the influence of inflammation. Trends Cardiovasc Med 33:357–366 35181472 10.1016/j.tcm.2022.02.005

[34] Ponnian SMP (2022) Preventive effects of (–) epicatechin on tachycardia, cardiac hypertrophy, and nuclear factor- κB inflammatory signaling pathway in isoproterenol-induced myocardial infarcted rats. Eur J Pharmacol 924:174909 35346644 10.1016/j.ejphar.2022.174909

[35] Dwaich KH, Al-Amran FGY, Al-Sheibani BIM, Al-Aubaidy HA (2016) Melatonin effects on myocardial ischemia–reperfusion injury: impact on the outcome in patients undergoing coronary artery bypass grafting surgery. Int J Cardiol 221:977–986 27441478 10.1016/j.ijcard.2016.07.108

[36] Raygan F, Ostadmohammadi V, Bahmani F, Reiter RJ, Asemi Z (2019) Melatonin administration lowers biomarkers of oxidative stress and cardio-metabolic risk in type 2 diabetic patients with coronary heart disease: a randomized, double-blind, placebo-controlled trial. Clin Nutr 38:191–196 29275919 10.1016/j.clnu.2017.12.004

[37] Javaherforoosh Zadeh F, Janatmakan F, Shafaeebejestan E, Jorairahmadi S (2021) Effect of melatonin on Delirium after on-pump coronary artery bypass graft surgery: a Randomized Clinical Trial. Iran J Med Sci 46:120–127 33753956 10.30476/ijms.2020.82860.1146 PMC7966933

[38] Chen L, Tian Q, Shi Z, Qiu Y, Lu Q, Liu C (2021) Melatonin alleviates cardiac function in Sepsis-caused myocarditis via maintenance of mitochondrial function. Frontiers in Nutrition 8. 10.3389/fnut.2021.754235 PMC8542660 34708067

[39] Colunga Biancatelli RML, Berrill M, Mohammed YH, Marik PE (2020) Melatonin for the treatment of sepsis: the scientific rationale. J Thorac Disease :S54–S65 10.21037/jtd.2019.12.85 PMC7024751 32148926

[40] Lv T, Yan J, Lou Y et al (2022) Evaluation of Melatonin Therapy in Patients with Myocardial Ischemia-Reperfusion Injury: A Systematic Review and Meta-Analysis. Oxidative Medicine and Cellular Longevity 2022:4610522 10.1155/2022/4610522 PMC8913055 35281465

[41] Ivry M, Goitein D, Welly W, Berkenstadt H (2017) Melatonin premedication improves quality of recovery following bariatric surgery – a double blind placebo controlled prospective study. Surg Obes Relat Dis 13:502–506 27979371 10.1016/j.soard.2016.11.001

[42] Galley HF, Lowes DA, Allen L, Cameron G, Aucott LS, Webster NR (2014) Melatonin as a potential therapy for sepsis: a phase I dose escalation study and an ex vivo whole blood model under conditions of sepsis. J Pineal Res 56:427–438 24650045 10.1111/jpi.12134 PMC4279949

[43] Mansilla-Roselló A, Hernández-Magdalena J, Domínguez-Bastante M et al (2023) A phase II, single-center, double-blind, randomized placebo-controlled trial to explore the efficacy and safety of intravenous melatonin in surgical patients with severe sepsis admitted to the intensive care unit. J Pineal Res 74:e12845 36428216 10.1111/jpi.12845 PMC10078138

[44] Menczel Schrire Z, Phillips CL, Chapman JL et al (2022) Safety of higher doses of melatonin in adults: a systematic review and meta-analysis. J Pineal Res 72:e12782 34923676 10.1111/jpi.12782

